# Commentary on alignment of medical student assessment and vocational training in psychiatry and addiction medicine

**DOI:** 10.1177/10398562231153010

**Published:** 2023-01-29

**Authors:** Jeffrey CL Looi, Daniel Bonner, Paul A Maguire, Matthew Brazel, Philip Keightley, Rebecca E Reay, Michael Tedeschi

**Affiliations:** Academic Unit of Psychiatry and Addiction Medicine, 104822 The Australian National University School of Medicine and Psychology, Canberra Hospital, Canberra, ACT, Australia; Academic Unit of Psychiatry and Addiction Medicine, 104822 The Australian National University School of Medicine and Psychology, Canberra Hospital, Canberra, ACT, Australia; Academic Unit of Psychiatry and Addiction Medicine, 104822 The Australian National University School of Medicine and Psychology, Canberra Hospital, Canberra, ACT, Australia; Academic Unit of Psychiatry and Addiction Medicine, 104822 The Australian National University School of Medicine and Psychology, Canberra Hospital, Canberra, ACT, Australia; Academic Unit of Psychiatry and Addiction Medicine, 104822 The Australian National University School of Medicine and Psychology, Canberra Hospital, Canberra, ACT, Australia; Academic Unit of Psychiatry and Addiction Medicine, 104822 The Australian National University School of Medicine and Psychology, Canberra Hospital, Canberra, ACT, Australia; Academic Unit of Psychiatry and Addiction Medicine, 104822 The Australian National University School of Medicine and Psychology, Canberra Hospital, Canberra, ACT, Australia

**Keywords:** Entrustable professional activity, workplace-based assessments, CanMEDs, medical education, vocational training

## Abstract

**Objective:**

To comment upon the potential for alignment of medical student assessment and vocational specialist training through the RANZCP-CanMEDS model of Entrustable Professional Activities (EPAs) and Workplace-Based Assessments (WBAs). We discuss a specific *post hoc* example of such an alignment in an Australian graduate medical school in Psychiatry and Addiction Medicine.

**Conclusions:**

Vocational training models of assessment, such as the RANZCP specialist training program for psychiatrists, can potentially be mapped to medical student education in formative and summative assessment through CanMEDs-based EPAs and WBAs, to assist in transition to specialist training.

“A beginning is the time for taking the most delicate care the balances are correct.”

Frank Herbert — Dune

Medical student education aims to prepare graduates for vocational training as junior medical officers, and thence, as medical specialists. There is potential for alignment of medical student assessment to existing processes used in vocational specialist training. Such alignment may facilitate transition from medical school education to specialty training. Particularly, we consider the educational concepts of Entrustable Professional Activities (EPAs)^1^ and Workplace-Based Assessments (WBAs),^2^ in the CanMEDS competency-based training model^3^ which has been substantively adopted for vocational specialist training in psychiatry in Australia and New Zealand.^4^ These concepts are also relevant for medical student education as they address skills, knowledge, and competencies that are foundational for specialist training. There has been substantial interest and activity on adapting the competency-based framework for EPAs and WBAs in health professional education,^5^ and specifically, in medical student education.^6^ There is evidence that EPA supervisor ratings for medical students correlate well with other summative assessments.^7^ There has been less research into the competency-based framework in Psychiatry and Addiction Medicine teaching, which was found to represent only 10% of studies in a previous systematic review.^6^ However, there is burgeoning development of such models in psychiatry teaching.^8^ Recent US research found there was a lack of medical student education alignment with post-graduate junior medical resident education and vocational training, particularly in the domain of EPAs.^9^ We therefore present a commentary on the alignment of medical student education with vocational specialist training in psychiatry in Australia.

## Mapping medical school education in Psychiatry and Addiction Medicine to CanMEDS competencies as a foundation for specialist training

CanMEDS encompasses a number of outcome-assessed medical practitioner competencies, including the central role as a medical expert, with specific overlapping domains as a Scholar, Professional, Communicator, Collaborator (with other health professionals and patients), Leader, and Health Advocate, adopted by a number of specialist colleges.^3^

For specialist training in psychiatry in Australia and New Zealand, there are three stages after internship and usually another pre-vocational JMO year: basic: stage 1, the first 12 months; proficient: stage 2, the next 24 months; and finally, advanced training: stage 3, the last 24 months of subspecialist training. The fundamental skills we expect of medical students map to the CanMEDs Medical Expert concept as defined by the RANZCP,^4^ commensurate with the beginning level of an intern, prior to Stage 1 vocational training. Students are expected to be able to:

1. Conduct a comprehensive, empathic, and culturally appropriate psychiatric assessment with patients of all ages.
2. Demonstrate the ability to perform and report a comprehensive mental state examination, which includes cognitive assessment.
3. Demonstrate the ability to integrate relevant available information in order to formulate the patient’s clinical presentation and make a diagnosis according to ICD or DSM.
4. Develop, negotiate, implement, and evaluate outcomes of a comprehensive evidence-based bio-psycho-socio-cultural management plan and appropriately revise.
5. Demonstrate the ability to integrate and appropriately manage the patient’s physical health with the assessment and management of their mental health problems.
6. Demonstrate the ability to appropriately apply mental health and related legislation in patient care.

## EPAs and WBAs—Definitions as educational foundations for specialist training

We quote from the RANZCP curriculum:^1^

“EPAs are specialised tasks that trainees undertake to demonstrate their ability to perform competently with only distant (reactive) supervision. Each EPA consists of specific knowledge, skills and attitudes required by the task….”

EPAs are assessed through satisfactory completion of a number of Workplace-Based Assessments (WBAs), which the RANZCP have benchmarked at three WBAs for a particular EPA. For example, in the RANZCP program there are EPAs pertaining to the CanMEDS domain of medical expertise that may be evaluated through different types of WBAs.

The WBAs that have been approved by the RANZCP are listed alongside the medical school equivalents in Table 1.^2^ In medical schools, WBAs can be used to assess entrustment of EPAs.

**Table 1.** RANZCP WBA assessments and medical school assessment equivalents

RANZCP WBA assessment	Medical school assessment
Case-based discussion	Case-based discussion with clinical supervisor
Mini-Clinical Evaluation Exercise	Observed structured clinical examination
Professional presentation	Presenting a case history
Direct observation of procedural skills	Observation of student administering cognitive assessment task or mental state examination
Observed clinical activity	Observation of an interview of a patient

There is a need for a standardized approach to use of EPAs in the medical curriculum that should involve initial development, implementation, and validation.^6^ To date, such an approach has not yet been adopted in our medical school, and it is also advocated that there should be a coordinated and limited core of EPAs, including those for psychiatry and addiction medicine, across the entire medical curriculum.^6^

## A graduate medical school teaching program in psychiatry and addiction medicine

In this context, we present a *post hoc* example of how an Australian graduate medical school program might map curricula and assessments to CanMEDS competencies through the application of medical student level EPA and WBAs. Our graduate medical education program teaches 100 students Psychiatry and Addiction Medicine in the final year of a 4-year program, comprising:

1. Six weeks of clinical placements, guided by formative portfolio tasks that are potentially functionally equivalent to EPAs and WBAs.
2. Intercalated didactic and interactive workshop teaching.
3. Summative end of year online written examinations including multiple-choice and extended matching questions, assessing knowledge.
4. Two online clinical assessment Objective Structured Clinical Examinations (OSCEs), one to assess mental state examination skills equivalent to a WBA and another, an interview with a simulated patient or patient relative/carer, again potentially equivalent to a WBA.

The most recent systematic review of EPAs in medical student education recommended the formative use of a portfolio, analogous to that used in our program, prior to summative supervisor entrustment.^6^

## Clinical placement portfolio tasks as examples of formative EPAs and WBAs

The observed interview WBA task involves the formative assessment of a student interview of a patient by a clinical supervisor (psychiatrist or trainee), at least once per 3-week rotation (each clinical placement is 6 weeks in length). For example, this task, derived from the medical student Psychiatry and Addiction Medicine program handbook, includes the following:

• Eliciting the relevant content of the patient’s history, with a view to later presenting it to their clinical supervisor, and a mental state examination.
• Presentation to and discussion with supervisor, covering history of presenting complaint(s), past (medical and psychiatric) and personal/developmental history, mental state examination, diagnosis, differential diagnosis, and a brief discussion of key management issues.

This Observed Interview therefore potentially maps to the CanMEDs Medical Expert competency, assessing the components described in detail above, as well as other components. This task is equivalent to a WBA in the form of an Observed Clinical Activity, involving assessment history-taking process, content, mental state examination skills, data synthesis, and if applicable, a management plan.^2^ There is a structured supervisor feedback form for this task, and other WBA-equivalent tasks, analogous to that required for RANZCP specialist training.

Other formative tasks, performed once per 6-week clinical placement, assess specific aspects traversing various CanMEDS competencies and may be considered as equivalent to WBAs, as a Case-based Discussion (Carer and Family Interview), Direct Observation of Procedural Skills (administration of a structured cognitive assessment). or other, as appropriate.

These formative assessments are consistent with recent Swiss research that indicates EPAs such as “take a patient history,” along with “assess physical and mental status” and “document and present a clinical encounter” are useful for formative medical student assessments in psychiatry.^10^ Our formative WBAs notionally map to similar formative EPAs.

Innovatively, the Swiss researchers assessed medical students’ self-ratings on entrustment before and after their psychiatric training clerkship and found that there were improvements on self-rated entrustment, as well as supervisor ratings, from mainly psychiatry residents and allied health workers.^10^ This study also incorporated narrative feedback on WBAs, which provided detail on the improvement of specific skills.^10^ Such self-assessments and feedback are being considered in our curriculum.

## OSCEs as WBAs satisficing summative EPAs

The final barrier assessments in Psychiatry and Addiction Medicine at our medical school consist of two OSCEs that are WBA-equivalents to Mini-Clinical Evaluation Exercises, an observed interview with a simulated patient or patient relative/carer for 20 min, which is rated by the examiner according to specific marking criteria.

There is also the presentation of a mental state examination based on viewing a video vignette of an actor/s portraying a mental illness, including specifically designated questions from examiners relating to phenomenology, diagnosis, differential diagnosis, and a management plan, according to structured marking criteria.

The summative EPA of being able to perform a satisfactory psychiatric assessment, including a history mental state examination and a preliminary intern-level management plan, can potentially be assessed based upon both the above WBA equivalents.

## Discussion

We have described an Australian *post hoc* example of how the CanMEDS model of vocational specialist training might be aligned to graduate medical student education in psychiatry. Our brief literature review found burgeoning research supporting the use of EPAs/WBAs in medical student education and in psychiatry teaching. CanMEDS, EPAs, and WBAs offer a complementary framework to design medical education to provide a more streamlined transition to medical vocational training. There remains much research to be conducted on the mapping of curricula to the competency-based framework to develop EPAs and WBAs, on implementation of these formative and summative assessments, as well as evaluation of self-entrustment by students and supervisor ratings and correlation with other assessment methods. Medical students also have views and rights in relation to the implementation of EPAs and must be included in planning, implementation, and evaluation.^11^ Furthermore, the evaluation of the effectiveness of medical student education as preparation for pre-vocational and vocational training is itself a substantial separate issue.^9^
